# Varicella-Zoster Virus Infection in an Immunocompromised Patient With Seizure-Like Activity and Septic Shock: A Case Report

**DOI:** 10.7759/cureus.103835

**Published:** 2026-02-18

**Authors:** Bryce Ebersole, Justin Chu, Muhammad Durrani

**Affiliations:** 1 Emergency Medicine, Inspira Medical Center, Vineland, USA

**Keywords:** aids, encephalitis, meningitis, seizure, shingles, varicella-zoster virus, vzv

## Abstract

Varicella-zoster virus (VZV) is well-known for causing primary chickenpox infection; however, it remains dormant in the central nervous system and can be reactivated during periods of decreased cell-mediated immunity. In addition to shingles, which is the most common form of reactivation, other rare secondary complications have been observed, including meningitis and encephalitis. Immunocompromised patients, such as those with acquired immunodeficiency syndrome (AIDS), are at an increased risk of such complications. We present the case of a 58-year-old male with AIDS (CD4 111 cells/mm^3^) who developed fevers, seizure-like activity, and septic shock. A lumbar puncture performed in the emergency department found the patient to have VZV infection. This case underscores a rare but devastating diagnosis that emergency physicians should be aware of.

## Introduction

The varicella-zoster virus (VZV) is a human alpha-herpesvirus that causes varicella or chickenpox during primary infection [1]. After primary infection, the virus lies dormant in ganglionic neurons near the spinal cord or brainstem and reactivates during periods of diminished VZV-specific cell-mediated immunity. Typically, the virus travels forward along the nerve and causes the classic vesicular “shingles” rash. Reactivation is more common in elderly patients and those who are immunocompromised due to acquired immunodeficiency syndrome (AIDS) or organ transplantation [2]. However, the virus may also travel backwards toward the brain and spinal cord, affecting the central nervous system (CNS).

The most common complication of VZV reactivation is the formation of a vesicular rash (shingles) following a dermatomal distribution and the sequelae associated with shingles [3]. VZV may also result in CNS complications such as meningitis, encephalitis, cerebritis, vasculopathy, and myelopathy. VZV meningitis-encephalitis is a rare but potentially life-threatening CNS infection defined by the presence of an inflammatory process of the meninges (meningitis) or brain (encephalitis) in association with clinical evidence of neurologic dysfunction [4].

Immunocompromised patients are at a higher risk for VZV infections, and being immunocompromised appears to be associated with poor outcomes [4]. Although the true incidence is unknown, a recent systematic review estimated a relative incidence of 0.02-2.7% in the general population [5]. The most commonly encountered symptoms are confusion followed by headaches, nausea, gait disturbances, and personality changes [6]. Factors that predict unfavorable outcomes include higher body temperature at presentation, a longer duration between CNS symptoms and start of antiviral therapy, higher white blood cell counts and adenosine deaminase in the cerebrospinal fluid (CSF), and higher serum C-reactive protein [4]. Frequently, patients with CNS infection also have a herpetic rash, which can aid the clinician in making the diagnosis [7]. This is, however, not always the case, as we highlight below. VZV meningitis-encephalitis is associated with high morbidity and mortality. Mortality rates at one and three months are noted to be 9% and 11%, respectively, in cohort studies [6]. Early identification of this condition and prompt treatment with intravenous antiviral medication are essential to avoid known complications. This case report serves to reinforce the body of literature on VZV meningitis-encephalitis and outlines the urgency of diagnosis and treatment even with atypical presentations.

## Case presentation

**Figure 1:**
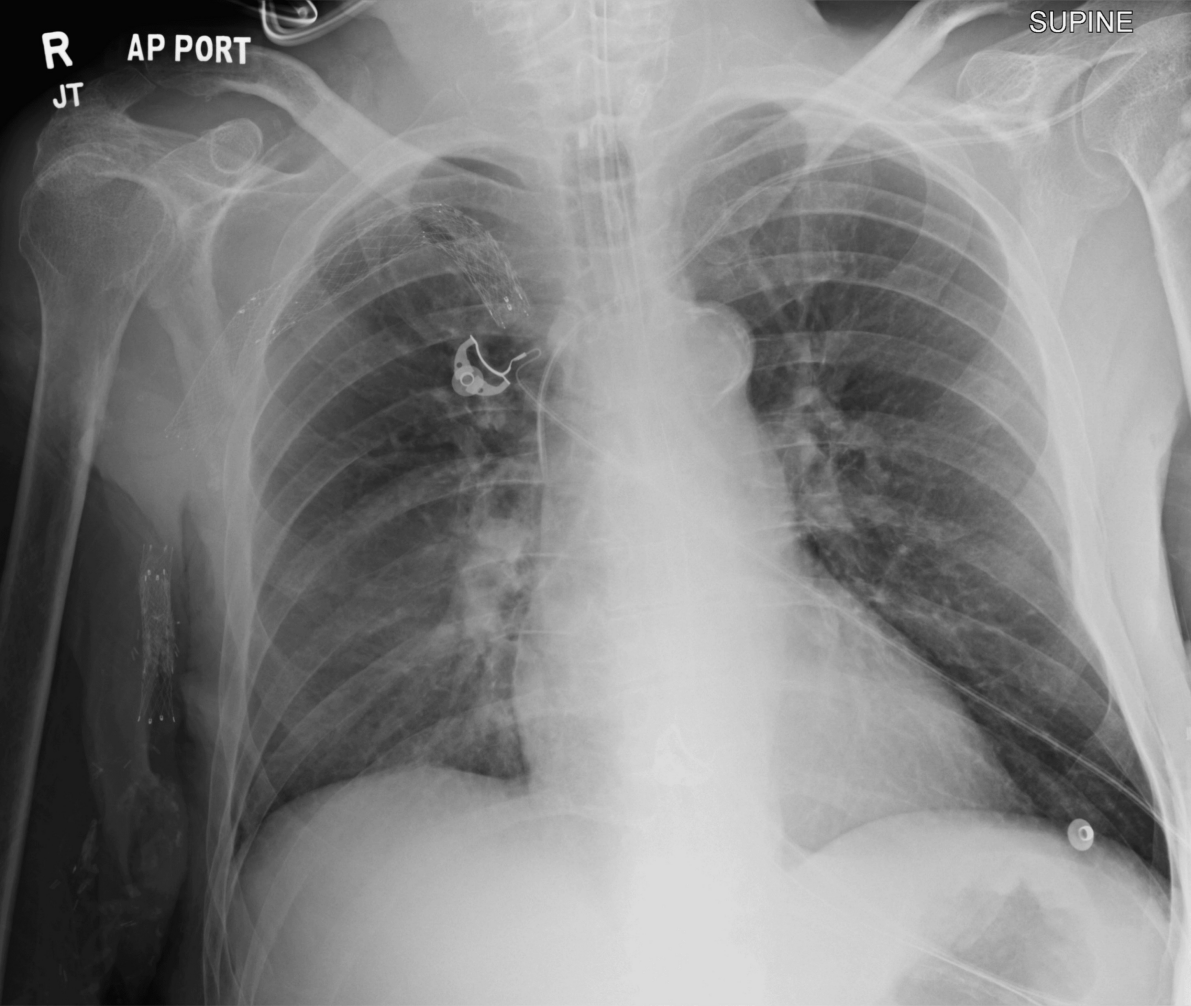
Chest X-ray showing clear lungs. The heart is normal in size. The endotracheal tube is seen with the tip 3.3 cm above the carina. The nasogastric tube is seen with the tip in the stomach.

A 58-year-old male with a past medical history of AIDS with a CD4 count of 111 cells/mm^3^ (maintained on dolutegravir 50 mg-rilpivirine 25 mg oral daily), end-stage renal disease on hemodialysis (on epoetin alfa 7,250 units subcutaneous daily), and Parkinson’s disease (maintained on carbidopa-levodopa 25-100 mg oral daily, pramipexole 0.5 mg oral daily, entacapone 200 mg oral daily) was brought in by emergency medical services (EMS) with an acute onset of confusion, respiratory distress, and seizure-like activity. The patient had a reported fever earlier in the day, according to EMS. EMS had administered 5 mg intravenous midazolam for the seizure-like activity and placed the patient on oxygen supplementation. On arrival, the patient was obtunded and ill-appearing with a Glasgow Coma Scale (GCS) score of 3. The patient’s vital signs revealed a blood pressure of 65/56 mmHg (reference range: systolic 90-120 mmHg, diastolic 60-90 mmHg), heart rate of 145 beats per minute (reference range: 60-100 beats per minute), rectal temperature of 39°C (reference range: 36.1-37.9°C), and an oxygen saturation of 65% (reference range: 92-100%). The patient underwent endotracheal intubation for airway protection using etomidate and rocuronium. Given his hypotension, tachycardia, and febrile state, he was treated with 30 cc/kg crystalloid infusion and administered broad-spectrum antibiotics (vancomycin 1,250 mg IV and cefepime 2 g IV as initial doses). Laboratory workup included a complete blood count, comprehensive metabolic profile, coagulation profile, blood cultures, serum lactic acid, high-sensitivity troponin, respiratory viral panel, and urinalysis. Diagnostic workup included an EKG, chest X-ray, and CT of the brain without contrast. The urinalysis and the portable chest X-ray showed no obvious signs of infection (Figure 1).

**Figure 2:**
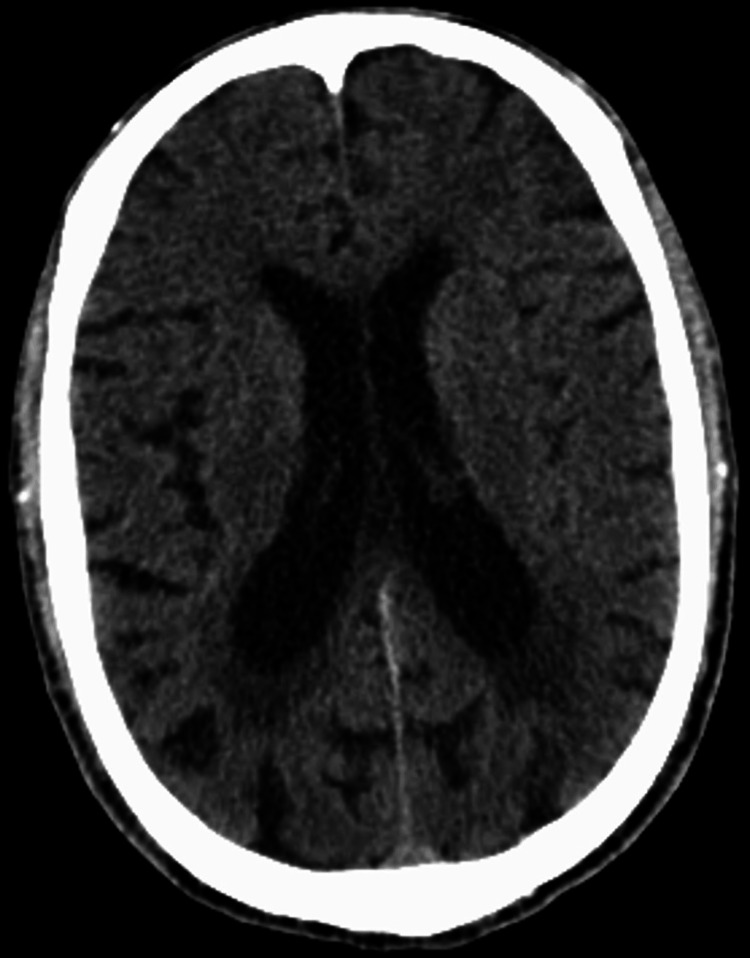
Non-contrast CT of the brain with no acute intracranial abnormality, intracranial hemorrhage, or mass effect. Age-appropriate volume loss and moderate white matter small vessel ischemic changes can be noted.

The patient remained hypotensive and was started on norepinephrine. Given the patient’s undifferentiated shock, febrile illness, and seizure activity, intravenous acyclovir 5 mg/kg (renal dosing) was started for empiric coverage of viral meningitis, and a lumbar puncture was performed. The patient’s CT of the brain was unremarkable (Figure 2).

**Figure 3:**
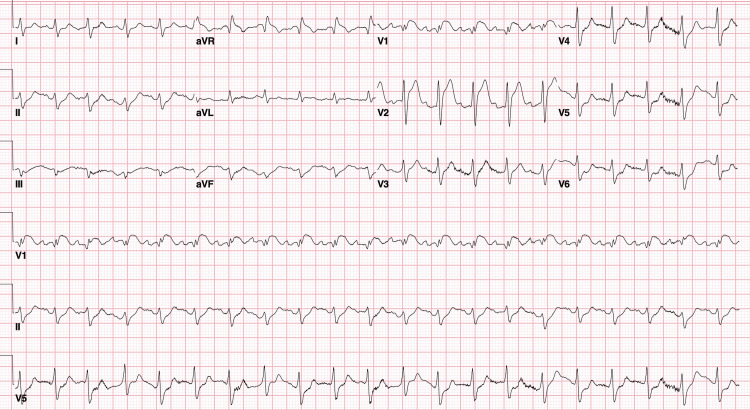
EKG revealing sinus tachycardia, ST depressed in inferior and lateral leads, and type 1 brugada pattern with ST elevation in leads V1 and V2.

An additional complicating factor in this patient’s presentation was the presence of ST-segment elevations in the septal leads of his initial EKG, with ST depression noted in the inferno-lateral leads, concerning for possible ST-elevation myocardial infarction (Figure 3).

**Figure 4:**
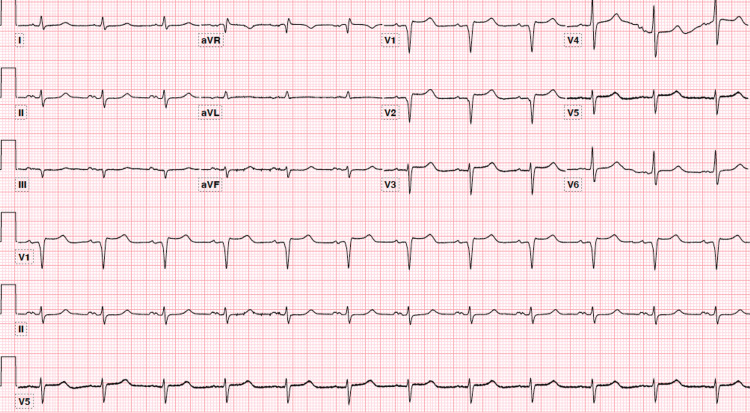
Repeat EKG showing normal sinus rhythm, left axis deviation, and prolonged qt.

Interventional cardiology was consulted, and serial EKGs were obtained (Figure 4).

Intervention cardiology did not believe that this EKG met the ST-elevation myocardial ischemia criteria; therefore, the emergent activation of the catheterization lab was not done. The initial high-sensitivity troponin level was 300 ng/L, and the subsequent level was 5,838 ng/L. Cardiology believed that the patient’s troponin elevation was likely due to demand mismatch caused by severe hypoxia versus undifferentiated shock, but that it was reasonable to start the patient on a heparin infusion pending further workup. Further diagnostics with a 2D echocardiogram and non-emergent cardiac catheterization were planned.

**Figure 5:**
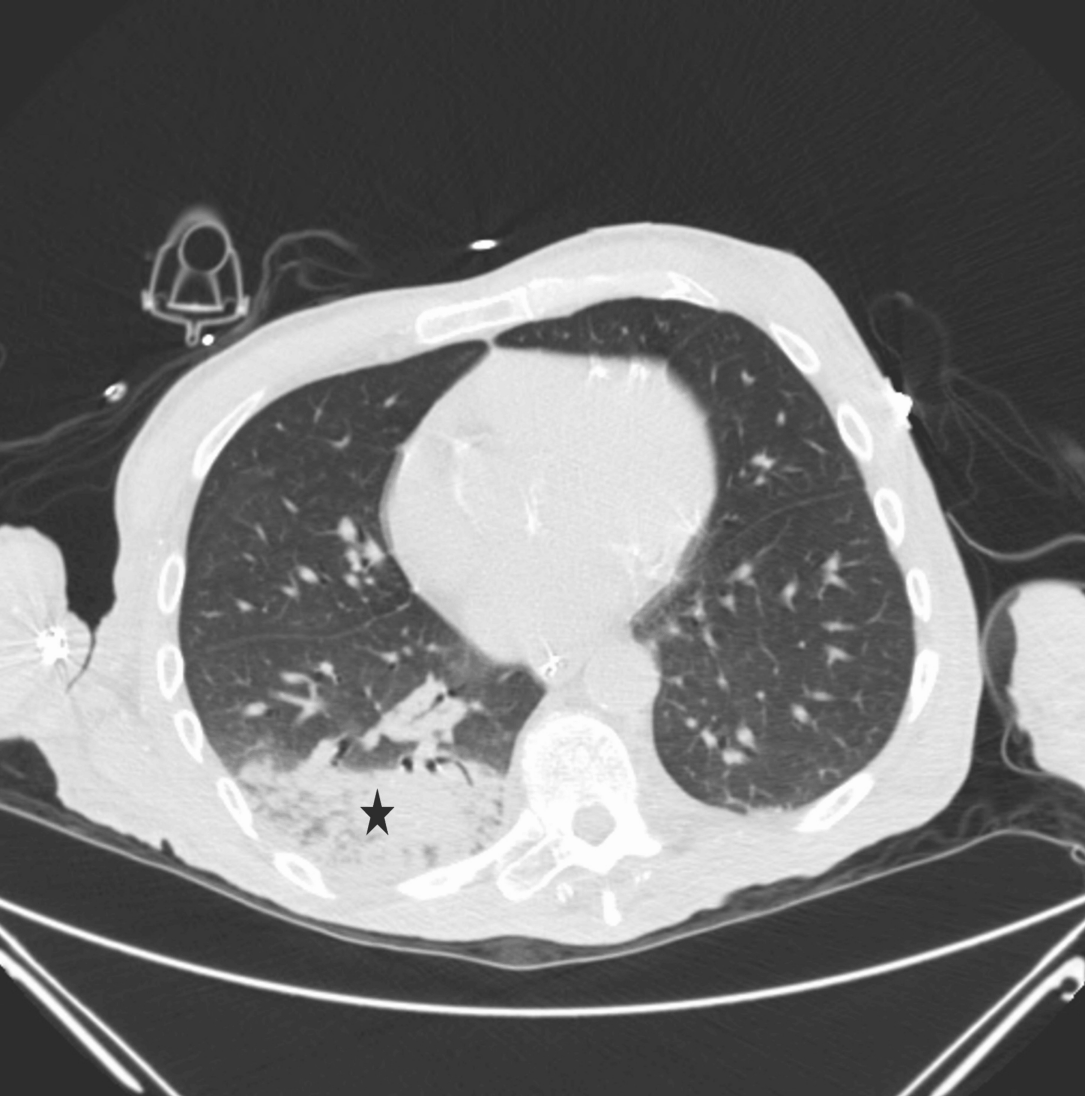
Non-contrast CT of the chest showing large dense consolidation (black star) of the right lower lobe.

CSF studies obtained from the lumbar puncture were consistent with viral meningitis with CSF nucleated cells at 0.027 × 10^3^/µL (reference range: 0-0.005 × 10^3^/µL) with 15% neutrophils (reference range: 0-6%), 57% lymphocytes (reference range: 40-80%), and 28% monocytes (reference range: 15-45%). Additionally, CSF studies revealed CSF protein at 246 mg/dL (reference range: 12-60 mg/dL), CSF glucose at 33 mg/dL (reference range: 40-70 mm/dL, serum glucose at the time of lumbar puncture at 84 mg/dL, and CSF polymerase chain reaction (PCR) studies were positive for VZV (Tables 1, 2). The patient was continued on daily acyclovir 5 mg/kg during his hospitalization. Additionally, before admission, the patient also underwent a CT of the thorax without contrast (obtained an hour after the initial chest X-ray in the emergency department), showing evidence of right lower lobe pneumonia (Figure 5).

**Table 1:** Cerebrospinal fluid studies.

Lab parameter	Value	Reference range
Appearance	Clear	Clear
Color	Colorless	Colorless
Nucleated cells (× 10^3^/µL)	0.027	0.000–0.005
Red blood cells (× 10^6^/µL)	<0.002000	<1.000000
Neutrophils (%)	15.0	0.0–6.0
Lymphocytes (%)	57.0	40.0–80.0
Monocytes (%)	28.0	15.0–45.0

**Table 2:** Additional lab values including complete blood count, basic metabolic panel, and high-sensitivity troponin.

Lab parameter	Value	Reference range
White blood cell (× 10^3^/µL)	11.8	4.0–11.0
Red blood cell (× 10^6^/µL)	6.47	3.80–4.70
Hemoglobin (g/dL)	14.9	12.5–17.0
Hematocrit (%)	48.7	37.0–50.0
Platelets (× 10^3^/µL)	199	140–380
Glucose (mg/dL)	84	74–106
Blood urea nitrogen (mg/dL)	36	9–23
Creatinine (mg/dL)	6.18	0.70–1.30
Sodium (mmol/L)	139	136–145
Potassium (mmol/L)	4.4	3.5–5.1
Chloride (mmol/L)	98	98–107
CO_2_ (mmol/L)	29.4	22.0–30.0
High-sensitivity troponin baseline (ng/L)	300	<3
High-sensitivity troponin at 2 hours (ng/L)	5,838	<3
Lactic acid (mmol/L)	2.5	0.5–2.2

During the patient’s admission, he was initially continued on broad-spectrum antibiotics until day five. His blood cultures as well as urine cultures showed no growth, and his sputum culture was positive for Methicillin-sensitive *Staphylococcus aureus*. Infectious disease believed his right-sided pneumonia noted on the CT scan of his chest was likely aspiration, and his antibiotics were changed to intravenous cefazolin 1 g daily for a planned five-day course. The patient was continued on acyclovir for a planned 14-day course. Unfortunately, he remained in shock, requiring vasopressors, and was unable to be weaned off the ventilator. The patient’s power of attorney and family collectively decided not to pursue tracheostomy or percutaneous endoscopic gastrostomy in light of prolonged mechanical ventilation. On day eight of intensive care unit admission, his power of attorney elected to compassionately extubate the patient, and he died.

## Discussion

This case highlights a rare but potentially devastating diagnosis (VZV meningoencephalitis) that emergency physicians should be aware of. In this instance, there was a high index of suspicion given the presentation of fever, altered mentation, and possible new-onset seizure in an individual with an immunocompromised state. One particularly challenging aspect of this case was the difficulty in determining if the patient had been showing seizure activity as opposed to rigors or even tremors associated with his Parkinson’s disease. However, there should be a low threshold for obtaining a lumbar puncture in the emergency department if clinical suspicion of meningoencephalitis is high, as in this case, looking for lymphocytic pleocytosis, increased red blood cells and neutrophils, as well as VZV DNA and antibodies by PCR [2]. Additionally, it is crucial to start empiric antiviral therapy as soon as possible when this diagnosis is being considered, as delays in treatment are associated with significant morbidity and mortality [8]. This is consistent with the Infectious Diseases Society of America guidelines that recommend early and empiric treatment to decrease death or serious sequelae associated with this disease process [9]. In this case, the patient was empirically started on acyclovir in the emergency department as the definitive workup was ongoing. The patient remained critically ill after admission, and, ultimately, he was unable to be weaned off of vasopressors and failed numerous spontaneous breathing trials in the intensive care unit. His power of attorney and family ultimately decided to pursue comfort measures, and he died on day eight of intensive care unit admission.

One of the more challenging aspects of the emergency department is that individuals are often working with limited information, whether that be a patient’s medical history or a history of illness leading up to the presentation. This diagnostic challenge is often compounded when there is an atypical presentation of a disease process, such as in this case. In this case, there was relatively limited information regarding the patient’s recent symptomatology leading up to his presentation, other than a fever that day. Thankfully, the team had ultimately decided to perform a lumbar puncture and start antiviral therapy in the emergency department, but several complicating factors in this case could have led to the diagnosis being missed with early diagnostic closure, including a diagnosis of septic shock with hypoxia, as well as EKG changes with elevated troponin. Studies have shown that sepsis and VZV CNS infection can be present concurrently, and there may also be a role of sepsis-induced immunosuppression leading to VZV reactivation [10]. Therefore, the presence of sepsis itself should not lead to early diagnostic closure without consideration of VZV infection in select patient populations [10]. Another factor that made this diagnosis challenging was that the patient did not have a rash on examination, even after being completely exposed in the emergency department. Historically, it has been noted that it is uncommon to have neurological sequela of VZV without cutaneous manifestations [3,11]. However, with newer advancements in diagnostic modalities, the diagnosis of intracranial VZV without skin lesions is becoming more common [3]. Therefore, an absence of a characteristic rash should not lead to early diagnostic closure.

In this clinical case, there was no previous history of epilepsy or documented seizure activity in the patient’s chart, and he did not appear to be on any antiepileptic medications at baseline. Moreover, the patient’s immunization history was also unavailable when he presented to the emergency department. EMS did report that they had witnessed seizure-like episodes before arrival at the emergency department. Additionally, in the emergency department, he appeared to have possible seizure-like activity; however, he was already intubated at this point, limiting a thorough neurological examination. Furthermore, history was limited secondary to his presenting mental status and GCS score upon arrival to the emergency department. Ultimately, it was the patient’s seizure-like activity that prompted consideration of meningoencephalitis as a potential diagnosis. Theoretically, his seizure-like activity could have also been rigors or Parkinsonian tremors, given his presenting fever and his history of Parkinson’s disease. Intravenous benzodiazepines did appear to improve these abnormal movements temporarily, and he required several doses of midazolam in the emergency department. Additionally, he was initiated on broad-spectrum antibiotics and received fluid resuscitation to cover for potential bacterial etiologies. However, maintaining a broad differential diagnosis and awareness of the possibility of CNS infection is what prompted empiric antiviral therapy and subsequent lumbar puncture in this patient.

Although rare, it is crucial to be aware of this disease process and its varied manifestations to promptly identify and treat it. The emergency physician should keep meningoencephalitis in mind when a patient presents with these constellations of symptoms, even if there are concurrent medical conditions.

## Conclusions

VZV is known to cause neurologic sequelae in reactivation, including meningoencephalitis. The emergency physician needs to remain vigilant when dealing with an immunocompromised patient who presents with signs of CNS infection, even in the absence of other clues such as a herpetic rash. These patients are typically very ill and may have other comorbid conditions such as bacterial pneumonia and sepsis, as this case highlights. We should have a low threshold to obtain a lumbar puncture and CSF studies in ruling out rarer complications in the immunocompromised patient population.

## References

[1] Nagel MA, Gilden D (2013). Complications of varicella zoster virus reactivation. Curr Treat Options Neurol.

[2] Gilden D, Nagel MA, Cohrs RJ, Mahalingam R (2013). The variegate neurological manifestations of varicella zoster virus infection. Curr Neurol Neurosci Rep.

[3] Kleinschmidt-DeMasters BK, Gilden DH (2001). The expanding spectrum of herpesvirus infections of the nervous system. Brain Pathol.

[4] Yuan Y, Wang J, Zhang Y, Liu H, Yan Y (2022). Factors predictive of varicella zoster virus encephalitis/meningitis: a single-center, retrospective study. Med Sci Monit.

[5] Giannelos N, Curran D, Nguyen C, Kagia C, Vroom N, Vroling H (2024). The incidence of herpes zoster complications: a systematic literature review. Infect Dis Ther.

[6] Nagel MA, Niemeyer CS, Bubak AN (2020). Central nervous system infections produced by varicella zoster virus. Curr Opin Infect Dis.

[7] Lenfant T, L'Honneur AS, Ranque B (2022). Neurological complications of varicella zoster virus reactivation: prognosis, diagnosis, and treatment of 72 patients with positive PCR in the cerebrospinal fluid. Brain Behav.

[8] Erdem H, Cag Y, Ozturk-Engin D (2015). Results of a multinational study suggest the need for rapid diagnosis and early antiviral treatment at the onset of herpetic meningoencephalitis. Antimicrob Agents Chemother.

[9] Tunkel AR, Glaser CA, Bloch KC (2008). The management of encephalitis: clinical practice guidelines by the Infectious Diseases Society of America. Clin Infect Dis.

[10] Miry L, De Mesmay M, Quirins M, Wemmert C, Khemili M, Engrand N (2024). Case report: varicella-zoster virus encephalitis a new type of "ICU-acquired infection"?. Heliyon.

[11] Nguyen A, Bauler L, Hoehn C, Mastenbrook J (2020). Varicella zoster virus meningoencephalitis with an atypical presentation of chest pain, impaired memory, and seizure. J Emerg Med.

